# Efficient Sustained-Release Nanoparticle Delivery System Protects Nigral Neurons in a Toxin Model of Parkinson’s Disease

**DOI:** 10.3390/pharmaceutics14081731

**Published:** 2022-08-18

**Authors:** Qun Wang, Rui Ma, Piaoxue Liu, Guowang Cheng, Qi Yang, Xiaojia Chen, Zhenfeng Wu, Dongsheng Yuan, Tongkai Chen

**Affiliations:** 1 Science and Technology Innovation Center, Guangzhou University of Chinese Medicine, Guangzhou 510405, China; 2 Key Laboratory of Modern Preparation of Traditional Chinese Medicine, Ministry of Education, Jiangxi University of Chinese Medicine, Nanchang 330004, China; 3 State Key Laboratory of Quality Research in Chinese Medicine, Institute of Chinese Medical Sciences, University of Macau, Macau 999078, China

**Keywords:** polymeric nanoparticles, blood-brain barrier, drug delivery, pharmacokinetics, brain accumulation, parkinsonian therapy

## Abstract

Parkinson’s disease (PD) is a serious neurodegenerative disease wherein the progressive destruction of dopaminergic neurons results in a series of related movement disorders. Effective oral delivery of anti-Parkinson’s drugs is challenging owing to the blood-brain barrier (BBB) and the limited plasma exposure. However, polymeric nanoparticles possess great potential to enhance oral bioavailability, thus improving drug accumulation within the brain. In this work, biodegradable poly(ethylene glycol)-b-poly(trimethylene carbonate) (PEG-PTMC) nanoparticles (PPNPs) were developed to deliver Ginkgolide B (GB) as a potent treatment for PD, aiming to enhance its accumulation within both the blood and the brain. The resultant GB-PPNPs were able to facilitate sustained GB release for 48 h and to protect against 1-methyl-4-phenylpyridine (MPP^+^)-induced neuronal cytotoxicity without causing any toxic damage. Subsequent pharmacokinetic studies revealed that GB-PPNPs accumulated at significantly higher concentrations in the plasma and brain relative to free GB. Oral GB-PPNP treatment was also linked to desirable outcomes in an animal model of PD, as evidenced by improvements in locomotor activity, levels of dopamine and its metabolites, and tyrosine hydroxylase activity. Together, these data suggest that PPNPs may represent promising tools for the effective remediation of PD and other central nervous system disorders.

## 1. Introduction

Parkinson’s disease (PD) is among the most prevalent forms of progressive neurodegenerative disease, causing serious morbidity and adverse socioeconomic impacts, particularly among elderly individuals [1]. Current treatments for PD include dopamine receptor agonists, the dopamine precursor levodopa, and monoamine oxidase B inhibitors [2]. However, these treatments only alleviate certain PD-related symptoms and fail to fully arrest disease progression or to remediate extant disabilities. Therefore, it is crucial that new and effective non-invasive treatments for PD are developed [3].

Ginkgolide B (GB) is a diterpene derived from the leaves of the *Ginkgo biloba* that is commonly considered to be a valuable neuroprotective drug with potential utility for the treatment of PD [4]. Notably, GB can interfere with the degeneration of the activity of tyrosine hydroxylase (TH), which is the rate-limiting dopamine-producing enzyme [5]. As such, GB treatment can protect against 6-hydroxydopamine-induced neurotoxic cell death among dopaminergic neurons. GB and Ginkgo biloba extract are also used clinically for the treatment of dementia and ischemic stroke [6,7]. However, GB water solubility is very poor and it exhibits very low bioavailability when administered orally, preventing it from accumulating at high levels in the systemic circulation and brain, thereby impeding its clinical anti-Parkinsonian utilization [8,9,10]. Polymeric nanoparticles are promising tools for drug delivery because they are biocompatible, biodegradable, and exhibit prolonged circulation [11,12]. Specifically, poly(ethylene glycol) (PEG) and poly(trimethylene carbonate) (PTMC) polymers are well-known FDA-approved biodegradable materials that are commonly utilized in a range of pharmaceutical and other medical contexts [13,14]. PEG-PTMC copolymers are amphiphilic and can form structures of varying molecular weights based upon the specific hydrophilic PEG and hydrophobic PTMC subunits employed [15]. Nanoparticles less than 100 nm in size have previously been reported to facilitate efficient drug delivery across the BBB [10,16]. Endocytosis followed by transcytosis are the underlying mechanisms for the BBB transport of these small-sized nanoparticles. Nanoparticle platforms possessing a prolonged, gradual drug release are of particular interest in the treatment of chronic diseases such as PD [17]. We herein sought to develop small PPNPs with gradual release characteristics capable of enhancing the oral bioavailability of GB and its accumulation within the brain tissues. To that end, an antisolvent precipitation approach was employed to encapsulate GB within PEG-PTMC, thus yielding GB-PPNPs. D-tocopheryl polyethylene glycol succinate (TPGS)-coated nanoparticles have previously been shown to be particularly effective tools for drug delivery across the BBB [18], since TPGS acts as a P-glycoprotein (P-gp) inhibitor [19]. However, the specific mechanisms governing the endocytic processing of these nanoparticles are not well understood. In this study, we additionally utilized Madin–Darby canine kidney (MDCK) cells as an in vitro model of the intestinal epithelium [20] because they are polarized cells that exhibit a thin mucus layer and tight junctions, such as those found in vivo, enabling the more reliable study of PPNPs endocytosis. We further used coumarin 6 (C6) to label PPNPs, a commonly used fluorescent to study how PPNPs penetrate biological barriers in zebrafish [21].

The main goal of this work was to develop the potential application of GB-PPNPs as mediators of sustained GB release and enhanced GB bioavailability, and to explore the ability of these PPNPs to enhance disease-related outcomes in a model of PD. Therefore, we characterized the endocytic processing of GB-PPNPs in cells and zebrafish, detected the pharmacokinetics of these PPNPs in rats, and evaluated their neuroprotection in an in vivo MPTP-induced PD model system. Through these analyses, we ultimately concluded that GB-PPNPs improved the oral bioavailability, brain accumulation, and therapeutic efficacy of GB.

## 2. Methods

### 2.1. Materials, Reagents, Cell Lines, and Animals

GB (purity ≥ 98%), C6 (purity ≥ 98%), and Levodopa (L-DOPA, purity ≥ 98%) were obtained from J&K Scientific Ltd. (Beijing, China). MPTP-HCl was obtained from MedChemExpress (South Brunswick Township, NJ, USA). 3-(4,5-dimethylthiazol-2-yl)-2, 5-diphenyltetrazolium bromide (MTT), 1-methyl-4-phenylpyridinium ion (MPP^+^) and rabbit polyclonal anti-TH were obtained from Sigma-Aldrich (St. Louis, MO, USA). PEG-PTMC was supplied by Jinan Daigang Biomaterial Co., Ltd. (Jinan, China). TPGS was obtained from Shanghai Yuanye Bio-Technology Co., Ltd. (Shanghai, China).

Two different cell models (MDCK and SH-SY5Y cells) were used in vitro. Both cultures were regularly maintained in a 5% CO_2_ incubator at 37 °C in DMEM supplemented with 10% FBS and 1% penicillin/streptomycin [22].

Adult wild-type zebrafish (*Danio rerio*) were raised under a 14 h light/10 h dark cycle to maturity, at which time male and female zebrafish were combined at a 1:2 ratio in a 1 L tank the night before breeding, separated by a mesh screen. Fertilized embryos were collected during the following light cycle, and all subsequent analyses were conducted at 28.5 °C using E3 medium [23].

Sprague–Dawley rats (male rats, 6–8 weeks) and C57BL/6 mice (male mice, 8 weeks) were obtained from the Experimental Animal Center of Guangzhou University of Chinese Medicine (Guangzhou, China), and were housed in a climate-controlled facility with free food and water access. The research was conducted in accordance with all guidelines and ethics of the Chinese Council on Animal Care.

### 2.2. GB-PPNP and C6-PPNP Preparation and Characterization

All PPNPs were prepared via antisolvent precipitation [24]. For GB-PPNPs, GB (20 mg/mL) and PEG-PTMC (20 mg/mL) in acetone was rapidly injected into TPGS (0.1 mg/mL in water) while stirring at 100× *g*. C6-PPNPs were prepared via an identical approach, with C6 being substituted for GB and with all procedures being performed in the dark. The resultant PPNPs were then characterized to assess the size distributions, polydispersity index (PDI) values, and zeta potential via dynamic light scattering (DLS). Each preparation was conducted in triplicate and each sample was detected in triplicate at room temperature. While morphology was assessed via transmission electron microscopy (TEM). Briefly, a droplet of the GB-PPNPs was carefully placed on a membrane-coated grid surface with a filter paper. The samples were negatively stained with phosphotungstic acid (2%, *w*/*v*) for 30 s. In addition, PPNP drug loading (DL) and encapsulation efficiency (EE) were measured via high-performance liquid chromatography. Briefly, samples (20 μL) were added to the high-performance liquid chromatography system (an auto-sampler, DAD detector, and analytical column) containing methanol/water (50:50), and were measured at 220 nm. Drug loading (DL) and entrapment efficiency (EE) were calculated as follows [25]:

$$\mathrm{DL}=\frac{\mathrm{weight}\;\mathrm{of}\;\mathrm{GB}\;\mathrm{in}\;\mathrm{GB}\text{-}\mathrm{PPNPs}}{\mathrm{weight}\;\mathrm{of}\;\mathrm{GB}\text{-}\mathrm{PPNPs}}\times 100\%$$

$$\mathrm{EE}=\frac{\mathrm{weight}\;\mathrm{of}\;\mathrm{GB}\;\mathrm{in}\;\mathrm{GB}\text{-}\mathrm{PPNPs}}{\mathrm{initial}\;\mathrm{weight}\;\mathrm{of}\;\mathrm{GB}}\times 100\%$$

During the in vitro drug release, GB-PPNPs or GB were monitored in phosphate-buffered saline (PBS, pH 7.4) by the dialysis method [9]. The system is maintained at a constant temperature of 37 °C and 100 rpm while stirring. Samples were collected at 0.5, 1, 2, 4, 6, 8, 10, 12, 24, and 48 h for the determination of GB content.

### 2.3. Assessment of GB-PPNP Uptake and Transport

MDCK cells were chosen as an in vitro model to investigate the cellular uptake and transport of GB-PPNPs. The cytotoxicity of GB-PPNPs or GB was assessed in vitro using MDCK cells via MTT assay. The uptake of these nanoparticles and free GB by MDCK cells was then assessed, and the apparent permeability coefficient (*P*_app_) was calculated to measure the permeability of GB, mixtures of GB and TPGS (GB-PM), and GB-PPNPs across an MDCK cell monolayer. Transepithelial electrical resistance (TEER) was assessed before and after such transformation to verify the integrity of the monolayer [26]. For further details, see Supporting Information, Section S1.

### 2.4. Evaluation of the Neuroprotective Efficacy of GB-PPNPs

Nerve cells (SH-SY5Y cells) were used to investigate the neuroprotective efficacy of GB-PPNPs. The ability of GB-PPNPs to improve nerve cell (SH-SY5Y) survival was assessed via MTT assay. In this study, SH-SY5Y cells were cultured in 96-well plates (5 × 10^3^/well) for 24 h, followed by treatment for 4 h with a range of GB or GB-PPNP concentrations and treatment for 24 h with MPP^+^ (2 mM, 10 μL per well), after which MTT (2 mM, 10 μL per well) was added for an additional 4 h. Absorbance at 570 nm was then assessed via a microplate reader to calculate the rate of cell survival [27].

### 2.5. Assessment of GB-PPNP Toxicity Using Zebrafish Embryos

At 3 h post-fertilization (hpf), zebrafish embryos were treated with 50, 100, 200, and 400 μg/mL GB-PPNPs (*n* = 20 per well). At 96 hpf, embryo morphology was visualized via microscopy, and survival rates, hatching rates, heart rates, and zebrafish body length were calculated [28].

### 2.6. Zebrafish Embryo and Larvae Imaging

At 3 hpf, zebrafish embryos were incubated with C6-PPNPs (50, 100, 200, or 400 ng/mL). After 5, 30, or 60 min, embryos were collected, rinsed with E3 medium, and assessed via fluorescence microscopy. Fluorescence images were obtained using a fluorescence microscope (Model DMi8, Leica, Germany). The microscope parameters were kept constant throughout the imaging process. Zebrafish C6-PPNP uptake at 7 days post-fertilization (dpf) was assessed via the same approach.

### 2.7. In Vivo Pharmacokinetic Analysis

To investigate the oral bioavailability and brain accumulation of GB-PPNPs, rats were randomly divided into two groups (GB-PPNPs and GB, GB dose of 4 mg/kg) and were then orally administered. Samples of serum were collected at appropriate time points for 0–48 h post-treatment (*n* = 7/time point). At each time point, blood (300 μL) was collected from the tail vein and centrifuged for 5 min at 2380× *g*, and supernatant serum was analyzed. In addition, brain samples were collected from rats at indicated time points post-treatment (*n* = 4/time point). Briefly, brains were perfused with physiological saline, removed, weighed, and homogenized in chilled saline. Drug contents in the biosamples were immediately measured via LC-MS/MS, as shown in the Supporting Information, Section S2.

Terminal elimination half-life (*T*_1/2_), area under the concentration-time curve from time zero to t (*AUC*_0-*t*_), time to maximum concentration (*T_max_*), peak concentration (*C_max_*), and mean residence time (*MRT*_0-*t*_) for the brain and plasma compartments were estimated using the Drug and Statistics (DAS, v 2.0, Shanghai Bojia Pharmaceutical Technology Co., Ltd., Shanghai, China) program with a non-compartmental model. Relative bioavailability (*F*) for the GB-PPNPs was assessed as follows:

$$F=\frac{AUC(\mathrm{GB}\text{-}\mathrm{PPNPs})}{\mathrm{AUCcontrol}}\times 100\%$$

### 2.8. In Vivo Pharmacodynamic Analysis

Mice were used to establish a PD model in this study due to the sensitivity of mice to MPTP. Mice were randomly assigned into five groups: (1) saline, (2) MPTP, (3) L-DOPA, (4) GB (5 mg/kg), and (5) GB-PPNP (5 mg/kg) groups. A murine PD model was established by intraperitoneally injecting mice in all groups (other than the control group) with 18 mg/kg of MPTP saline solution four times with 2 h between injections [29]. Groups (3) and (4) were orally administered GB or GB-PPNPs dispersion for two weeks in total, including once per day for one week before MPTP treatment and twice per day for one week thereafter. Group (1) was orally treated with saline, while animals in the L-DOPA group received intraperitoneal injections of L-DOPA (25 mg/kg). Behavioral testing, immunohistochemical staining for TH^+^ neurons, and levels of dopamine and metabolites were examined to assess the neuroprotective properties of these different treatments [30,31]. For further details, see Supporting Information, Section S3.

### 2.9. Histological Staining

At appropriate time points, mice were euthanized and major organs (lungs, kidneys, spleen, liver, heart) were fixed with 4% formalin, and then paraffin embedded sectioning was conducted for hematoxylin and eosin (H&E) to examine cellular damage and inflammation.

### 2.10. Statistical Analysis

Values were expressed as mean ± standard deviation (SD). The statistical differences between two groups were analyzed via unpaired two-tailed Student’s *t*-test. A one-way analysis of variance (ANOVA) was applied for more than two groups. For all tests, *p* < 0.05 was designated as the threshold for statistical significance.

## 3. Results and Discussion

### 3.1. GB-PPNP and C6-PPNP Preparation and Characterization

**Figure 1:**
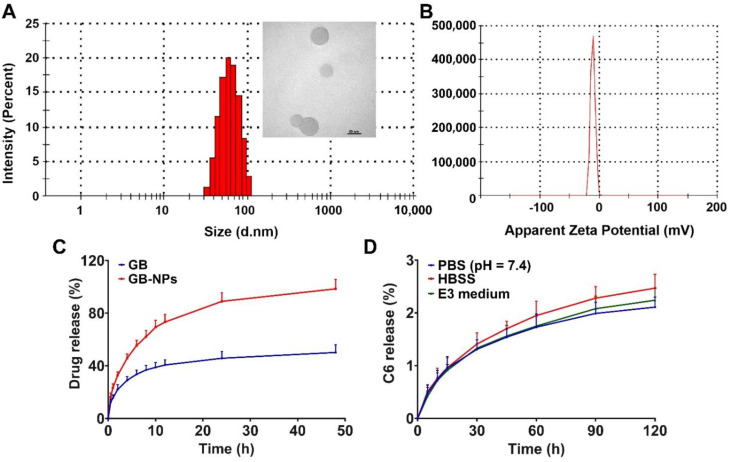
GB-PPNP and C6-PPNP characterization. (**A**) GB-PPNP size and TEM image. Scale bar: 50 nm. (**B**) GB-PPNP surface charge. (**C**) GB drug release from different formulations (means ± SD, *n* = 3). (**D**) Assessment of C6 leakage from C6-PPNPs in PBS, HBSS, and E3 medium (means ± SD, *n* = 4).

After preparation, GB-PPNPs exhibited an average particle size of 77.58 ± 0.77 nm, an average PDI of 0.124 ± 0.018 (Figure 1A), and a surface charge of −10.37 ± 0.56 mV (Figure 1B). These particles were spherical in morphology (Figure 1A), with a DL of 19.43% and an EE of 92.08%. They remained of uniform size and distribution even following a two-week incubation at room temperature (Figure S1). In this study, GB-PPNPs were prepared via antisolvent precipitation because of its low price and simplicity of operation with narrow particle size distribution and high drug loading [24]. In vitro drug release analyses performed using these particles in PBS (pH 7.4) revealed that GB was released from these particles in a biphasic manner (Figure 1C), with an initial rapid release over the first 4 h and then a slower phase in which sustained gradual release was detected over the remaining 48 h. This rapid burst release of GB at early time points is likely attributable to the free drug and drug adsorbed to the surfaces of these nanoparticles, whereas subsequent gradual release is more likely mediated via diffusion and dissolution. Compared to the free drug, the encapsulation of GB in PPNPs significantly improved its water solubility.

C6-PPNPs were prepared via the same antisolvent technique used to synthesize GB-PPNPs, and exhibited similar characteristics including a particle size of 75.91 ± 0.83 nm, a PDI of 0.181 ± 0.037 (Figure S2), and a surface charge of −11.65 ± 0.89 mV (Figure S3). C6 leakage from these PPNPs was then assessed in PBS, HBSS, and E3 medium to evaluate the utility of this compound as a marker for PPNP localization (Figure 1D). As under 3% of the loaded C6 leaked from these particles over a 2 h period, this suggested that C6 was effectively loaded so that it remained stably associated with the resultant PPNPs even under conditions of gradual intracellular acidification.

### 3.2. Evaluation of GB-PPNP Uptake and Permeability Characteristics Using MDCK Cells

GB-PPNP treatment was not associated with any MDCK cell cytotoxicity within the tested range (5–100 μM) in an MTT assay (Figure S4). Compared to the free drug (0.97 ± 0.09 μg/mg protein) and a physical mixture of TPGS and GB treatment (GB-PM; 1.03 ± 0.14 μg/mg protein), GB-PPNP uptake by MDCK cells was significantly enhanced (3.29 ± 0.97 μg/mg protein) without significant differences in uptake for the former two treatments. According to a previous report, we speculated that GB-PPNPs might be endocytosed via clathrin, and that the small particle size of GB-PPNPs made this process more accessible [32]. The *P*_app_ value for the GB-PPNP group (3.59 ± 0.32 × 10^−5^ cm/s) was also markedly higher than that for the GB group (1.14 ± 0.12 × 10^−5^ cm/s) or the GB-PM group (1.2 ± 0.14 × 10^−5^ cm/s), indicating that GB-PPNPs are more readily able to transit across the MCDK cell monolayer. TEER values did not significantly differ before or after treatment in any of these three groups, indicating that monolayer integrity was not adversely impacted.

### 3.3. GB-PPNPs Exhibit Neuroprotective Efficacy When Used to Treat SH-SY5Y Cells

GB-PPNP treatment was similarly not associated with any SH-SY5Y cell toxicity within the tested range (1–200 μM) in an MTT assay (Figure S5). As an in vitro PD model system, MPP^+^ was used to treat SH-SY5Y, with a 2 mM MPP^+^ dose resulting in the death of 45.28% of the treated cells. When these cells were first pretreated with GB-PPNPs (1, 5, 10, or 20 μM), their viability was significantly improved following MPP^+^ exposure (57.63%, 66.22%, 77.2%, and 93.73%, respectively) (Figure S6). Notably, this effect was more pronounced than that observed for GB. The neuroprotective efficacy of GB-PPNPs might be attributed to the antioxidative stress and the activation of the protein kinase B (Akt)/glycogen synthase kinase-3β (Gsk3β) pathway of GB [5,33].

### 3.4. Analysis of GB-PPNP Toxicity in Zebrafish Embryos

**Figure 2:**
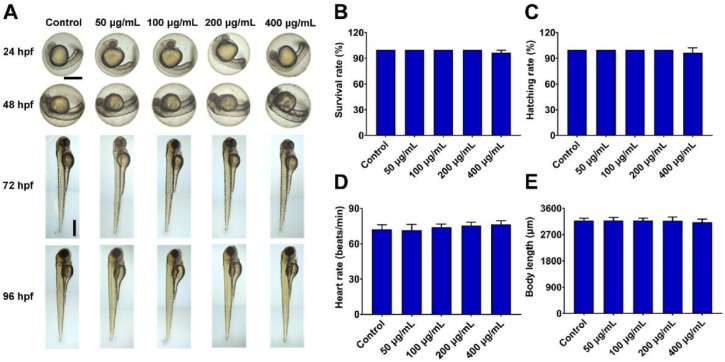
In vivo toxicity analysis. At 3 hpf, zebrafish embryos were treated with 50, 100, 200, and 400 μg/mL GB-PPNPs. At 96 hpf, embryo morphology was visualized via microscopy, and survival rates, hatching rates, heart rates, and zebrafish body length were calculated. (**A**) The phenotypic changes of GB-PPNP-treated zebrafish embryos at the indicated times. Scale bar: 500 μm. Survival rate (**B**), hatching rate (**C**), heart rate (**D**), and body length (**E**) following incubation with different GB-PPNP treatments (*n* = 3).

As zebrafish exhibit whole-body transparency, they serve as an ideal vertebrate model system for monitoring drug-related phenotypic and morphological changes [34,35]. Importantly, zebrafish also harbor biological barriers with significant structural and functional similarity to those found in humans [36] and they were thus used to evaluate the biocompatibility of PPNP preparations. In this study, zebrafish embryos (3 hpf) were treated with a range of GB-PPNP concentrations (50, 100, 200, and 400 μg/mL) and monitored for changes in development, blood flow, and visible malformations at the indicated times. No morphological abnormalities in zebrafish embryos or larvae were evident after GB-PPNP treatment (Figure 2A), and there were similarly no treatment-related changes in survival rates, hatching rates, heart rates, or body length at 96 hpf in any groups (Figure 2B–E). Therefore, GB-PPNPs do not induce significant toxicity in vivo in zebrafish, consistent with our in vitro cytotoxicity analyses.

### 3.5. Imaging of Zebrafish Embryos and Larvae

**Figure 3:**
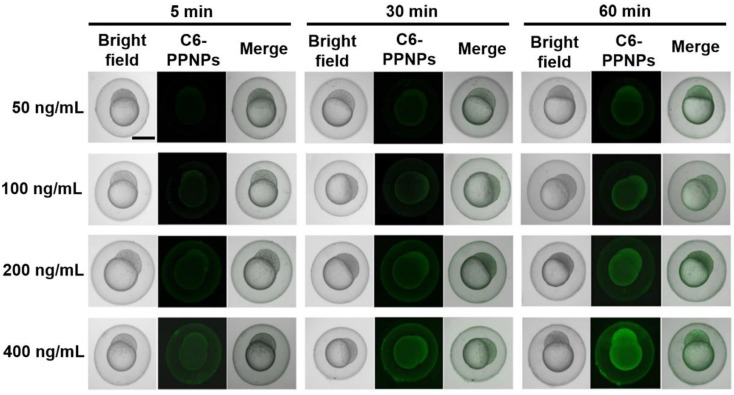
Imaging of zebrafish embryos. Zebrafish embryos (3 hpf) following incubation with C6-PPNPs at C6 concentrations of 50, 100, 200, and 400 ng/mL for 5, 30, and 60 min. Scale bar: 500 μm.

To examine in vivo GB-PPNP uptake, zebrafish were utilized as a small vertebrate model system, with C6 serving as a fluorescent dye to efficiently track PPNPs localization [37]. Zebrafish embryos (3 hpf) were treated with C6-PPNPs for a range of time periods in order to evaluate particle movement across the chorion [38]. The resultant fluorescence intensity increased in a dose- and time-dependent fashion from 5–60 min (Figure 3), suggesting that these small PPNPs were able to penetrate the chorion and accumulate in the yolk sac. These data suggest that GB-PPNPs improve the ability of drugs to pass through biological barriers.

**Figure 4:**
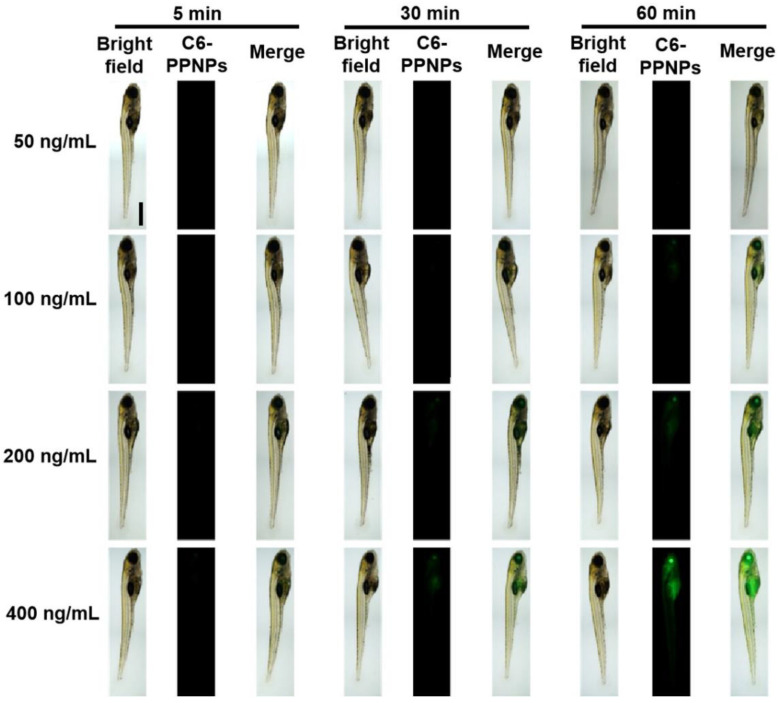
Imaging of zebrafish larvae. Zebrafish embryos (7 hpf) following incubation with C6-PPNPs at C6 concentrations of 50, 100, 200, and 400 ng/mL for 5, 30, and 60 min. Scale bar: 500 μm.

To further examine the ability of the prepared GB-PPNPs to cross the BBB and the gastrointestinal barrier in vivo, zebrafish (7 dpf) were exposed to C6-PPNPs, which did not induce significant toxicity as evidenced by the results shown in Figures S7–S11. Substantial fluorescent uptake was detectable in the brains and digestive system of these zebrafish (Figure 4), consistent with the ability of these orally absorbed C6-PPNPs to cross the gastrointestinal barrier and thereby enter the brain. A strong fluorescent signal was also evident in the eyes (Figure 4), consistent with crossing the blood-retinal barrier. These data thus provide further evidence that our PPNPs are able to readily pass through key physiological barriers. However, the BBB transport mechanism is required for further investigation.

### 3.6. In Vivo Pharmacokinetic Analysis

**Figure 5:**
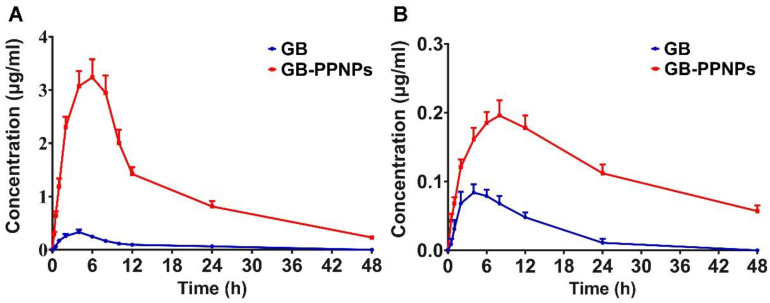
In vivo analysis of GB-PPNP pharmacokinetics. (**A**) Plasma curve (*n* = 7). (**B**) Brain curve (*n* = 4).

To understand the processing and trafficking of GB-PPNPs within a mammalian system, rats were next used to conduct a series of pharmacokinetic analyses assessing the plasma and brain levels of GB-PPNPs at various time points after administration. As presented in Figure 5A and Table 1, GB-PPNPs exhibited a *C_max_* of 3.24 ± 0.34 μg/mL, with this value being higher than that for the GB group (0.33 ± 0.05 μg/mL), suggesting that GB-PPNPs are readily and rapidly absorbed in vivo. Such absorption is likely attributable to the surface properties and particle sizes of these nanoparticles. In addition, these GB-PPNPs were slowly eliminated from the serum, with a *T*_1/2_ of 7.26 ± 0.68 h. The *T_max_* and *AUC*_0-*t*_ values in the GB-PPNP group (6.67 ± 1.03 and 54.62 ± 4.82, respectively) were higher than those in the GB group. Additionally, the *AUC*_0-*t*_ of GB following PPNP treatment was markedly higher than that reported by Liu et al. [9]. In line with these findings, we observed the *MRT*_0-*t*_ value for GB-PPNPs to be enhanced to 9.87 ± 1.11 h as compared to 8.71 ± 0.75 h for the GB group.

When the brain pharmacokinetics of GB-PPNPs were assessed (Figure 5B and Table 1), the *C_max_* and *AUC*_0-*t*_ in the GB-PPNP group (0.20 ± 0.02 μg/g and 5.66 ± 0.47 μg·h/g, respectively) were markedly higher than those in the GB group (0.08 ± 0.01 μg/g and 1.11 ± 0.15 μg·h/g, respectively). The increase of *C_max_* and *AUC*_0-*t*_ might be due to the desirable brain accumulation of these nanoparticles. The brain *T*_1/2_ treated with GB-PPNPs was evidently longer than that treated with GB nanocrystals (13.67 ± 1.07 h vs. 3.93 ± 0.29 h, respectively) [9]. The brain *T_max_* for GB-PPNPs was also somewhat higher than the plasma value (7.53 ± 1.22 h vs. 6.67 ± 1.03 h, respectively), suggesting that GB is eliminated from the brain more gradually than from systemic circulation. These results thus support the value of the TPGS stabilizer in these GB-PPNPs as a means of enhancing brain penetration following treatment, thus accounting for increased drug accumulation within the brain. As such, these pharmacokinetic data indicate that the GB-PPNPs may offer an effective approach to enhancing the absorption and intracerebral accumulation of GB in vivo.

### 3.7. In Vivo Pharmacodynamic Analysis

**Figure 6:**
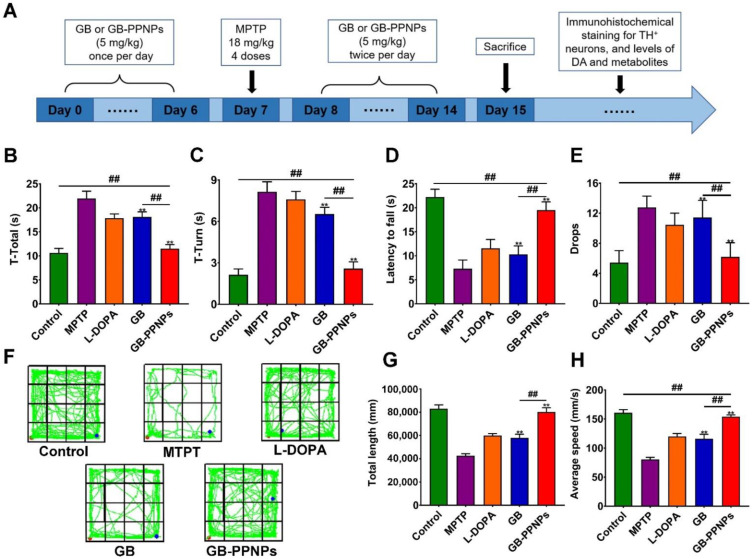
GB-PPNPs alleviate MPTP-induced behavioral impairments in PD model mice. (**A**) Pharmacodynamic study design. After MPTP injection, pole tests (**B**,**C**) and rotarod tests (**D**,**E**) were performed (*n* = 6). (**F**) Representative paths (green) for mice activity in the indicated groups. The red and blue dots represent the start and the end of the positions, respectively. Distance traveled (**G**) and average travel speed (**H**) for animals in the indicated groups (*n* = 8). ** *p* < 0.01 vs. MPTP. ^##^ *p* < 0.01 vs. GB-PPNPs.

In order to evaluate the efficacy of GB-PPNPs as a treatment for PD-related locomotor disorders, we performed a pharmacodynamic analysis (Figure 6A). MPTP treatment was used to establish a murine model of PD [39]. Pole and rotarod tests were used to assess delayed movement recovery and muscle coordination in these mice, while an open-field test was employed to assess their exploratory behavior [40]. Following MPTP treatment, model mice exhibited significant movement impairments, with significant reductions in the time spent on the rod together with significant increases in the time to turn and the total time (t-turn and t-total, respectively) (Figure 6B,C). However, GB-PPNP-treated mice exhibited significant reductions in t-total and t-turn values. Similarly, GB-PPNP-treated mice exhibited markedly increased fall latency and decreased numbers of falls relative to MPTP-treated model mice (Figure 6D–F). In an open-field test, GB-PPNP treatment resulted in increased speed and average travel distance relative to model mice (Figure 6F–H). These results indicated that GB-PPNPs were able to reverse MPTP lesion-related impairments in balance and coordination in vivo. However, the neuroprotection mechanism is required for further investigation.

**Figure 7:**
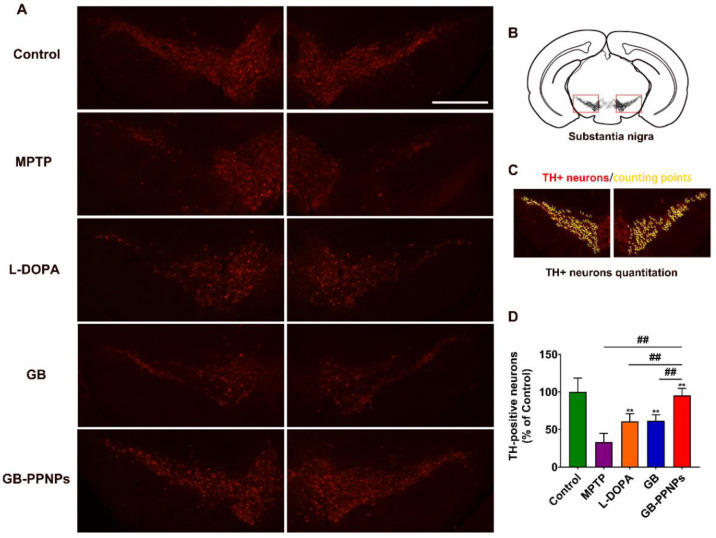
The in vivo neuroprotection of GB-PPNPs in MPTP-treated PD model mice. (**A**) Representative TH-stained murine brain sections. (**B**) Schematic illustration of the representative brain sections. Scale bar: 500 μm. (**C**) Quantification of TH^+^ neurons by manual counting with ImageJ software. (**D**) The number of TH^+^ neurons (both the right and the left substantia nigra) in the indicated treatments (*n* = 6). ** *p* < 0.01 with respect to MPTP. ^##^ *p* < 0.01 with respect to GB-PPNPs.

**Figure 8:**
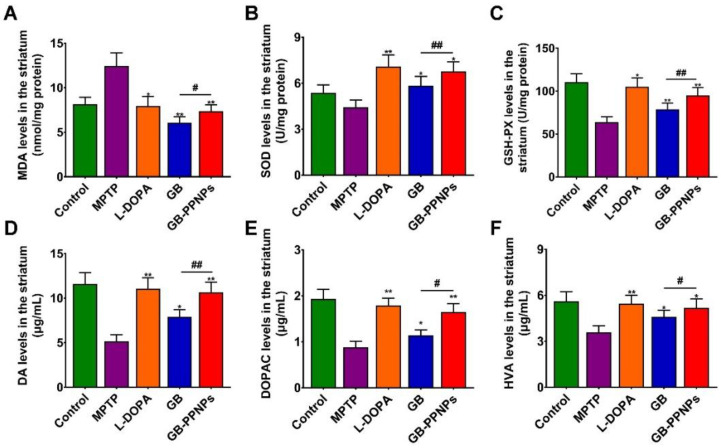
The impact of GB-PPNPs on striatal. The levels of (**A**) MDA, (**B**) SOD, (**C**) GSH-Px (means ± SD, *n* = 4), (**D**) dopamine, (**E**) DOPAC, and (**F**) HVA (*n* = 7). * *p* < 0.05 and ** *p* < 0.01 corresponds to different treatments vs. MPTP. ^#^ *p* < 0.05 and ^##^ *p* < 0.01 corresponds to GB-PPNPs vs. GB.

MPTP treatment was associated with the induction of significant dopamine neuron cell death so that few TH^+^ cells were detectable in the brains of treated mice [41]. However, GB-PPNP treatment was linked to an increase in the number of surviving dopamine neurons (Figure 7A,B), with this number rising significantly to 91.30% of the control as compared to 41.94% of the control in the MPTP model group (Figure 7C,D). MPTP-associated neurotoxicity was associated with altered dopamine metabolism, as evidenced by the fact that mice in the GB-PPNP treatment group exhibited striatal dopamine, DOPAC, and HVA concentrations of 10.66 ± 1.12, 1.65 ± 0.18, and 5.17 ± 0.60 μg/g tissue weight, respectively, with these values being significantly higher than those observed in the MPTP group (5.14 ± 0.73, 0.88 ± 0.13, 3.58 ± 0.42 μg/g tissue weight, respectively) (Figure 8).

Malondialdehyde (MDA) is an endogenous genotoxic substance produced by the lipid peroxidation of unsaturated fatty acids in phospholipids, which means that the increase of MDA is accompanied by oxidative stress [42]. Glutathione peroxidase (GSH-Px) and superoxide dismutase (SOD) are the main antioxidant enzymes in the human antioxidant system [43]. In this study, mice treated with GB-PPNPs also exhibited a striatal MDA level of 7.37 ± 0.70 nmol/mg protein, with this being lower than that in MPTP-treated mice (12.42 ± 1.48 nmol/mg) in the striatum (Figure 8). We further found that mice treated with GB-PPNPs exhibited higher striatal SOD and GSH-Px levels (6.77 ± 0.63 and 94.86 ± 9.15 U/mg protein, respectively) relative to MPTP model mice (4.42 ± 0.48 and 63.92 ± 6.06 U/mg protein, respectively) (Figure 8).

**Figure 9:**
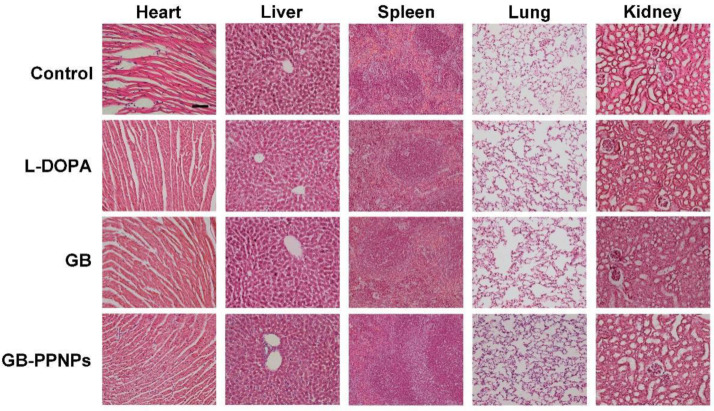
H&E-stained organs in the indicated groups. The scale bar is 50 μm.

No cellular damage or inflammation were observed in treated animals via H&E staining, indicating that treatment with GB-PPNPs (5 mg/kg) is both safe and effective (Figure 9).

## 4. Conclusions

In summary, we report spherical nanoparticles (77.58 ± 0.77 nm in diameter) composed of PEG-PTMC, TPGS, and GB as the carrier, stabilizer, and model drug, respectively. The resultant particles were highly stable, exhibited a negative surface charge, and caused negligible toxicity in a zebrafish model system. When C6 was utilized to track the in vivo fate of these PPNPs, they were found to readily cross the BBB and chorionic barrier in zebrafish. Pharmacokinetic studies performed in rats clearly revealed that GB-PPNPs exhibited enhanced brain uptake efficiency, as evidenced by higher plasma and brain GB concentrations in rats administered GB-PPNPs relative to animals dosed with free GB. In a murine model of MPTP-induced PD, GB-PPNP treatment alleviated behavioral deficits, attenuated dopaminergic neuron depletion, and enhanced the levels of dopamine, DOPAC, and HVA in analyzed samples. Together, these results provide robust evidence that GB-PPNPs can be utilized for the oral delivery of GB or other anti-Parkinson’s drugs in order to efficiently treat PD, owing to their enhanced ability to deliver drugs with poor oral bioavailability to the brain.

## Figures and Tables

**Table 1:** Plasma and brain pharmacokinetic parameters of GB-PPNPs following oral administration (*n* = 7 or 4). Significant differences between free GB and GB-PPNPs are marked with * for *p* < 0.05 and ** for *p* < 0.01.

Parameters	GB	GB-PPNPs
Plasma		
*T*_1/2_ (h)	2.07 ± 0.16	7.26 ± 0.68 *
*T_max_* (h)	3.33 ± 1.63	6.67 ± 1.03 *
*C_max_* (μg/mL)	0.33 ± 0.05	3.24 ± 0.34 *
*AUC*_0-*t*_ (μg·h/mL)	3.37 ± 0.34	54.62 ± 4.82 **
*MRT*_0-*t*_ (h)	8.71 ± 0.75	9.87 ± 1.11 **
F	100%	1621%
Brain		
*T*_1/2_ (h)	3.31 ± 0.36	13.67 ± 1.07 **
*T_max_* (h)	4.59 ± 1.04	7.53 ± 1.22 *
*C_max_* (μg/g)	0.08 ± 0.01	0.20 ± 0.02 *
*AUC*_0-*t*_ (μg·h/g)	1.11 ± 0.15	5.66 ± 0.47 **
*MRT*_0-*t*_ (h)	8.72 ± 0.81	18.90 ± 1.63 **

## Data Availability

The data presented in this study are available on request from the corresponding author. The data are not publicly available due to further studies in progress.

## Supplementary Materials

The following supporting information can be downloaded at: https://www.mdpi.com/article/10.3390/pharmaceutics14081731/s1, Figure S1: Stability of GB-PPNPs; Figure S2: Particle size distribution of C6-PPNPs; Figure S3: Zeta potential of C6-PPNPs; Figure S4: MDCK cell viability following treatment with the indicated GB and GB-PPNPs concentrations; Figure S5: SH-SY5Y cell viability following treatment with the indicated GB and GB-PPNPs concentrations; Figure S6: SH-SY5Y cell viability with the indicated GB and GB-PPNPs concentrations following MPP^+^ treatment; Figure S7: There were no morphological changes in zebrafish after treatment with different concentrations of C6-PPNPs; Figure S8: Body length of 96 hpf zebrafish treated with different concentration of C6-PPNPs; Figure S9: Survival rate of 96 hpf zebrafish treated with different concentration of C6-PPNPs; Figure S10: Heart rate of 96 hpf zebrafish treated with different concentration of C6-PPNPs; Figure S11: Hatching rate of 96 hpf zebrafish treated with different concentration of C6-PPNPs. References [22,44] are cited in the Supplementary Materials.
