# Supplementary material for: Efficient Sustained-Release Nanoparticle Delivery System Protects Nigral Neurons in a Toxin Model of Parkinson’s Disease
Source: Pharmaceutics. 2022 Aug 18;14(8):1731. doi: 10.3390/pharmaceutics14081731 (PMC9415969; doi:10.3390/pharmaceutics14081731)
Supplement: Supplementary file 1 [file pharmaceutics-14-01731-s001.zip › pharmaceutics-1842215-supplementary.pdf]

Supporting Information

Efficient Sustained-Release Nanoparticle Delivery System Protects Nigral Neurons in a Toxin Model of Parkinson’s Disease

Qun Wang [1,†], Rui Ma [1,†], Piaoxue Liu [1], Guowang Cheng [2], Qi Yang [3], Xiaojia Chen [3], Zhenfeng Wu [2,*], Dongsheng Yuan [1] and Tongkai Chen [1,*]

1 Science and Technology Innovation Center, Guangzhou University of Chinese Medicine, Guangzhou 510405, China
2 Key Laboratory of Modern Preparation of Traditional Chinese Medicine, Ministry of Education, Jiangxi University of Chinese Medicine, Nanchang 330004, China
3 State Key Laboratory of Quality Research in Chinese Medicine, Institute of Chinese Medical Sciences, University of Macau, Macau 999078, China
* Correspondence: zfwu527@163.com (Z.W.); chentongkai@gzucm.edu.cn (T.C.); Tel.: +86-791-8711-9032 (Z.W.); +86-20-3658-5707 (T.C.)
† These authors contributed equally to this work.

S Methods

S1 Cellular uptake and transport studies

S1.1 MTT assays

MDCK cells were grown to 70% confluency in DMEM supplemented with 10% FBS. Cells were then incubated at a density of 5000 cells/cm^2 in a 96-well plate for 24 h at 37 °C, 5% CO_2. After cells were exposed to various concentrations of GB or GB-PPNPs (1-200 μM) for 24 h, MTT (5 mg/mL) was added and incubated for 4 h, the medium was then removed and 150 μL DMSO was added to dissolve the formazan product. Relative cell viabilities were determined by the optical densities at 570 nm with a microplate reader.

S1.2 Cellular uptake in MDCK cells

MDCK cells were seeded at a density of 2 × 10^5 cells/mL in a 12-well plate

at 37 °C for 48 h. Subsequently, cells were treated with 20 μM of GB, GB-PPNPs or GB-PM. After incubation for 2 h, the cells were washed twice with sterile PBS and lysed. To assess the cellular uptake levels of GB, methanol was added to precipitate the protein, samples were vortexed for 2 min and subsequently centrifuged at 15,000× *g* for 20 min at room temperature. Supernatants were processed to determine the GB contents by HPLC.

S1.3 Transport of GB-NCs across MDCK cell monolayers

The permeability across MDCK cell monolayers was evaluated for GB-PPNPs, GB and GB-PM. To ensure cell monolayer integrity, the values of transepithelial electrical resistance (TEER) were measured before and after experiments. For measuring the apical-to-basolateral transepithelial transport, 600 μL of Hank's buffered salt (HBS, pH 7.4) solution containing of 20 μM GB, an equal concentration of GB-PPNPs or GB-PM was added to the apical side. Immediately thereafter, 100 μL of the solution was collected to determine the initial concentration (C_0). On the other hand, 1.5 mL of HBS solution was added to the basolateral side (receptor chamber). Cell monolayers were incubated at 37 °C for 2 h. After incubation, 200 μL of samples were collected from the basolateral compartments at 0.25, 0.5, 1, 1.5 and 2 h, and replaced with the same volume of fresh medium. The concentration of GB was detected using the HPLC method. The following Equation was used to calculate the apparent permeability coefficient, P_{app} (cm/s).

$$P_{app} = \frac{dC / dt \times V}{C_0}$$

where *dC/dt* represent the amount of drug transported over time (ng/mL·s), *V* is the volume (mL) of HBS solution at receptor chamber, *A* is the surface area (cm^2) of cell monolayers, and C_0 is the initial concentration (ng/mL) of HBS solution at the apical side.

S2 Samples extraction and LC-MS/MS analysis

100 μl rat plasma was mixed with 190 μl methanol, and then 10 μl methanol (containing 100 ng/mL chloramphenicol) was added as internal standard. The samples were tested by LC-MS/MS method. Brain tissue was homogenized with ultra turrax in 1:2 (G: ML) pre cooled saline for 2-3 minutes. After centrifugation at 9500x g for 10 minutes, the supernatant was collected and filtered for LC-MS / MS analysis. The samples were analyzed using a Q-Trap 4000 MS/MS system from Applied AB Sciex (Foster City, CA, USA) coupled to an Agilent 1100 series HPLC system (Santa Clara, USA). The chromatographic separation was performed on an Ecosil C18 column at room temperature. The mobile phase was methanol/water (50:50, V/V), and the pumping flow rate was 200 μL/min. The total running time of each sample was 8.5 min. The method of MS/MS was to monitor the m/z 423.1 → 367.1 fragment in GB and 321.0 → 152.0 fragment in internal standard chloramphenicol.

S3 In vivo pharmacodynamics

S3.1 Pole test

The pole test for bradykinesia was conducted in reference to a previous report [22], and performed at seven days post-MPTP administration. Mice were

placed head downward on the top of a vertical pole (1 cm in diameter and 50 cm in height) with a rough surface. Each mouse was placed head-up near the top of the pole and time to turn (t-turn) and time to reach the bottom (t-total) were recorded. For each mouse, the pole test was repeated three times.

S3.2. Rotarod test

Mouse was placed in a rotarod (diameter, 7 cm) at a fixed speed of 20 r/min in the 2 min testing. The time that each mouse spent on the rod (latency to fall) and the total drops were measured. Rotarod test was repeated three times for each mouse.

S3.3. Open-field test

Open-field test was carried out in an arena (60 cm × 60 cm × 40 cm), which was divided into 16 squares (15 cm × 15 cm). Before testing, each mouse was placed in the center of the arena for free exploration within 10 min. Then the total distances of moving and mean moving speed were recorded in the 15 min testing. The open-field test was repeated three times for each mouse. Results of the open-field test were analyzed using Flyde-maze software.

S3.4. Immunofluorescence

After perfusion with 4% paraformaldehyde, brains were removed, postfixed in the same solution at 4 °C overnight, cryoprotected in 30% sucrose, and finally 30 μm coronal sections were obtained according to a previous report [44]. In immunofluorescence detection, antigen retrieval was performed by 70 °C heating of the sections in sodium citrate buffer (10 mM trisodium citrate,

0.5% Tween-20 in H_2O, pH 6.0) for 30 min. The sections were blocked with 10% goat serum (with 0.5% Triton X-100) in Tris-buffered saline for 20 min and labeled with TH (Abcam, 1:1000) in blocking buffer over night at 4 °C. After that, the slides were washed with PBS for three times (5 min in each time), incubated with anti-rabbit secondary antibodies conjugated to Alexa Fluor 594 (Cell Signaling Technology, 1:1000) for 1 h. Thereafter, the slides were washed with PBS for three times again. Finally, the sections were covered with coverslips and mounted with anti-fade fluorescence mounting medium (Beyotime Biotechnology, Jiangsu, China) and observed under a fluorescence microscope (Model DMi8, Leica, Germany). The TH^+ neurons quantification was analyzed by counting the numbers of positive cells at ×200 magnifications on a fluorescence microscope (Model DMi8, Leica, Germany). Quantification of TH^+ neurons by manual counting with ImageJ software. Briefly, open the image by clicking on "File"→"Open", then select "Multi-Point tool", and mark every red fluorescence dot that represented TH^+ neurons cells in substantia nigra. Finally, perform analysis of the cell numbers.

S3.5 Measurement of dopamine and its metabolites in the striatum

The striatum was weighted and sonicated in 0.4 M $HClO_4$ (10 μL per milligram tissue) on ice and then centrifuged at 10,000 r/min for 10 min at 4 °C. The supernatants were removed for determining the concentration of DA and its metabolites DOPAC and HVA using the chromatograph (ESA, Chelmsford, MA, USA) with a 5014B electrochemical detector.

S3.6 Antioxidative activity evaluation by biochemical assay

To assess the changes of oxidative injury indexes, four mice of each group were euthanized and their substantial nigra were dissected out and homogenized in PBS buffer. The samples were then centrifuged at 10,000 r/min for 10 min at 4 °C. The protein assay in the supernatant was quantified by BCA protein assay kit. MDA, SOD and GSH assay kits were used for measurement according to the manufacturer instructions, respectively.

S Results

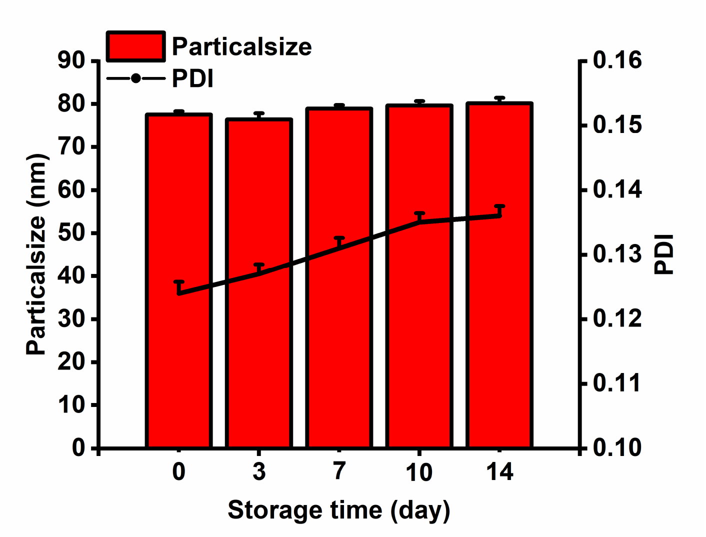

Figure S1. Stability of GB-PPNPs.

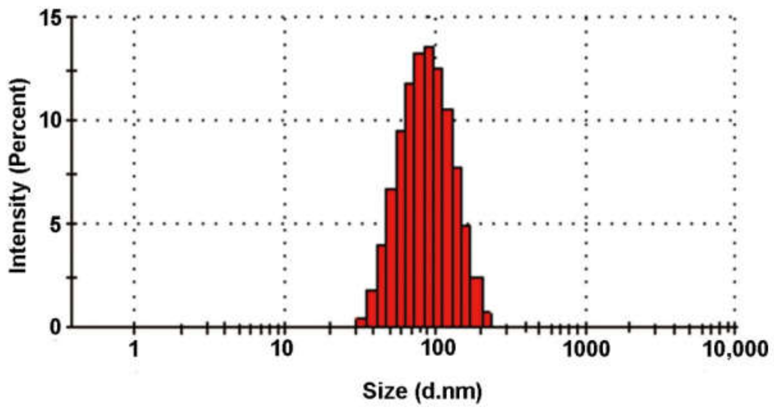

Figure S2. Particle size distribution of C6-PPNPs.

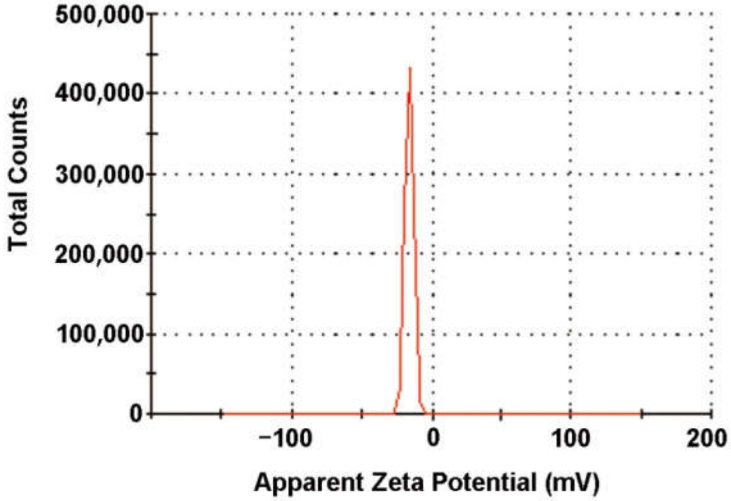

Figure S3. Zeta potential of C6-PPNPs.

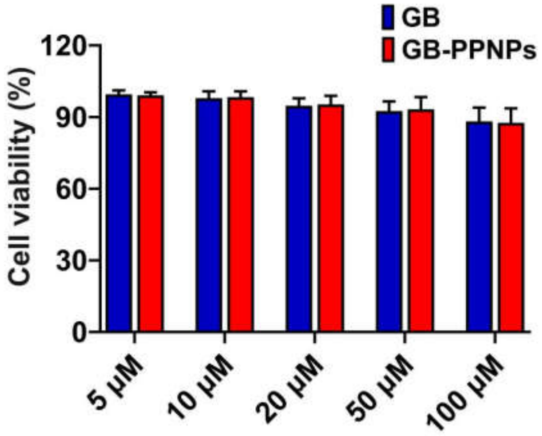

Figure S4. MDCK cell viability following treatment with the indicated GB and GB-PPNPs concentrations. The data are presented as means ± SD (n = 3).

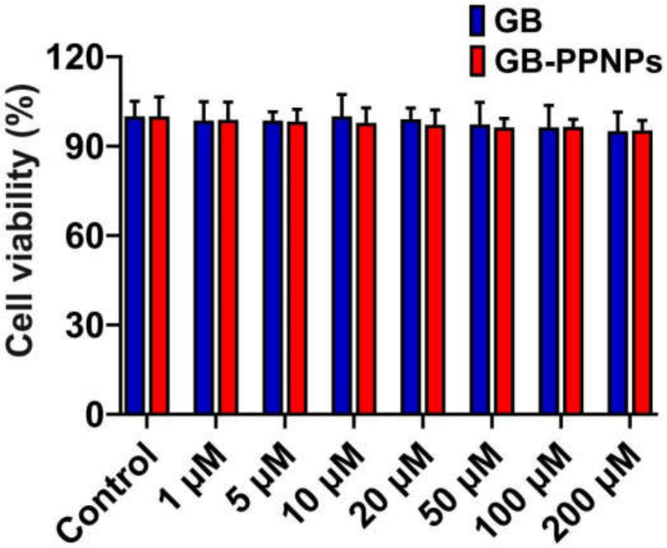

Figure S5. SH-SY5Y cell viability following treatment with the indicated GB and GB-PPNPs concentrations. The data are presented as means ± SD (n = 3).

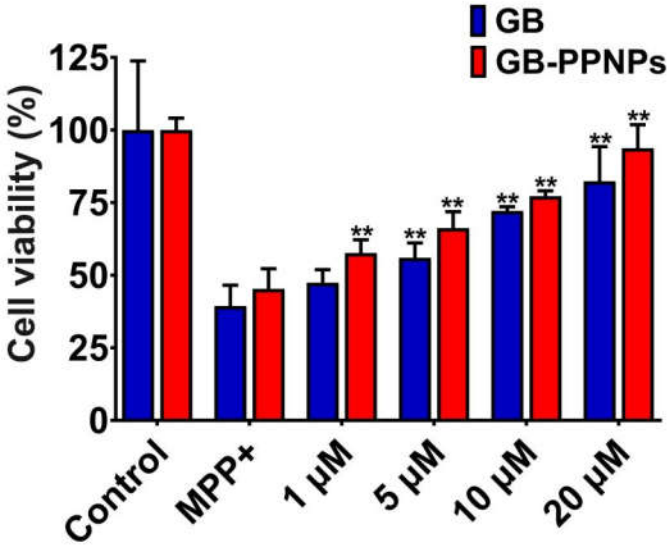

Figure S6. SH-SY5Y cell viability with the indicated GB and GB-PPNPs concentrations following MPP^+ treatment. Data are presented as means ± SD (n = 3). Relative to MPP^+ group: ** $p < 0.01$.

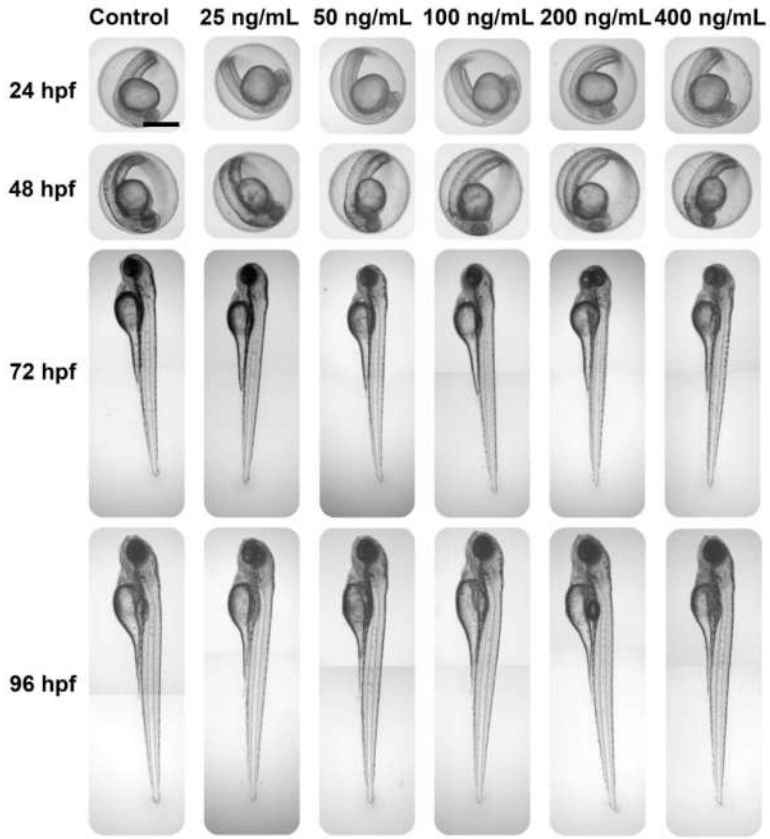

Figure S7. There were no morphological changes in zebrafish after treatment with different concentrations of C6-PPNPs. Scale bar: 500 μm.

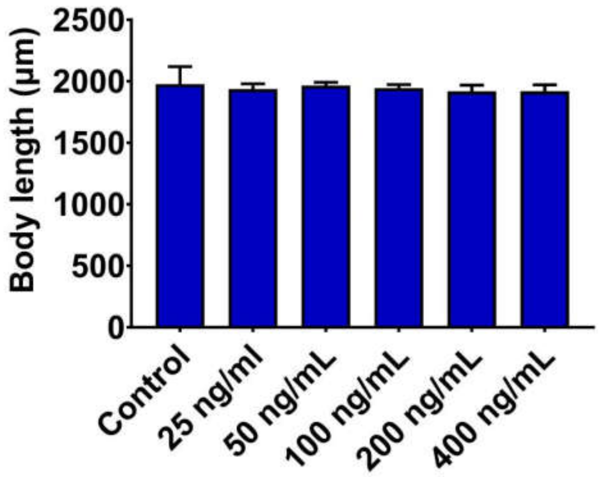

Figure S8. Body length of 96 hpf zebrafish treated with different concentration of C6-PPNPs.

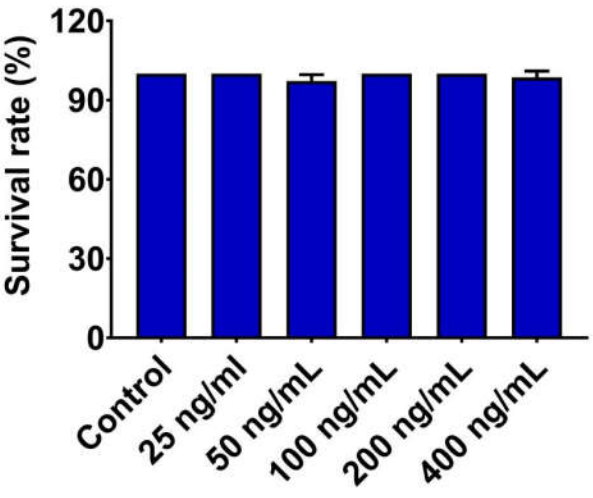

Figure S9. Survival rate of 96 hpf zebrafish treated with different concentration of C6-PPNPs.

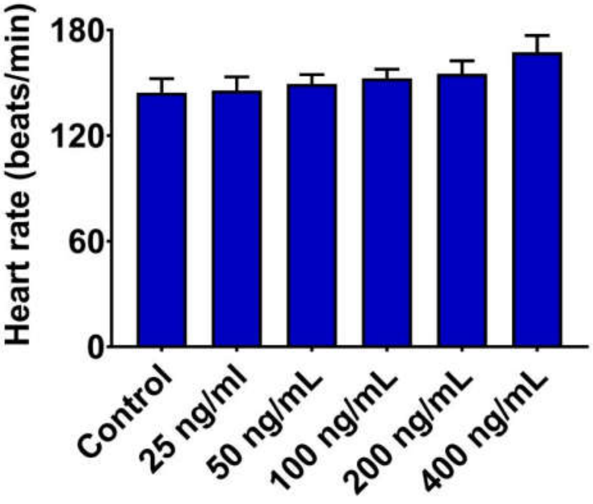

Figure S10. Heart rate of 96 hpf zebrafish treated with different concentration of C6-PPNPs.

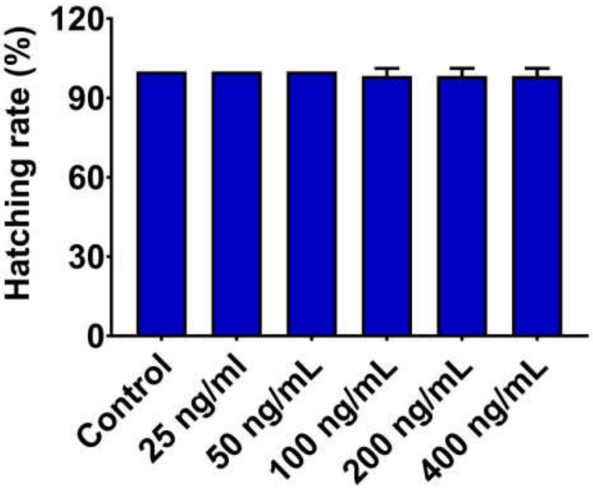

Figure S11. Hatching rate of 96 hpf zebrafish treated with different concentration of C6-PPNPs.

References

22. Liu, Y.; Zhu, D.; Luo, J.; Chen, X.; Gao, L.; Liu, W.; Chen, T. NIR-II-Activated Yolk-Shell Nanostructures as an Intelligent Platform for Parkinsonian Therapy. *ACS Appl. Bio Mater.* **2020**, *3*, 6876–6887, https://doi.org/10.1021/acsabm.0c00794.

44. O'Hara, D.; Kapadia, M.; Ping, S.; Kalia, S.; Kalia, L. Semi-Quantitative Determination of Dopaminergic Neuron Density in the Substantia Nigra of Rodent Models using Automated Image Analysis. *J. Vis. Exp. JoVE* **2021**, 168, e62062. https://doi.org/10.3791/62062.
